# CCW-YOLOv5: A forward-looking sonar target method based on coordinate convolution and modified boundary frame loss

**DOI:** 10.1371/journal.pone.0300976

**Published:** 2024-06-03

**Authors:** Yan Sun, Bo Yin

**Affiliations:** College of Information Science and Engineering, Ocean University of China, Qingdao, China; VIT-AP Campus, INDIA

## Abstract

Multi beam forward looking sonar plays an important role in underwater detection. However, due to the complex underwater environment, unclear features, and susceptibility to noise interference, most forward looking sonar systems have poor recognition performance. The research on MFLS for underwater target detection faces some challenges. Therefore, this study proposes innovative improvements to the YOLOv5 algorithm to address the above issues. On the basis of maintaining the original YOLOv5 architecture, this improved model introduces transfer learning technology to overcome the limitation of scarce sonar image data. At the same time, by incorporating the concept of coordinate convolution, the improved model can extract features with rich positional information, significantly enhancing the model’s detection ability for small underwater targets. Furthermore, in order to solve the problem of feature extraction in forward looking sonar images, this study integrates attention mechanisms. This mechanism expands the receptive field of the model and optimizes the feature learning process by highlighting key details while suppressing irrelevant information. These improvements not only enhance the recognition accuracy of the model for sonar images, but also enhance its applicability and generalization performance in different underwater environments. In response to the common problem of uneven training sample quality in forward looking sonar imaging technology, this study made a key improvement to the classic YOLOv5 algorithm. By adjusting the bounding box loss function of YOLOv5, the model’s over sensitivity to low-quality samples was reduced, thereby reducing the punishment on these samples. After a series of comparative experiments, the newly proposed CCW-YOLOv5 algorithm has achieved detection accuracy in object detection mAP@0.5 Reached 85.3%, and the fastest inference speed tested on the local machine was 54 FPS, showing significant improvement and performance improvement compared to existing advanced algorithms.

## Introduction

With the increasing demand of intelligent underwater detection in industrial and scientific research, the research of underwater sonar image target detection becomes more and more important. Whether in civil or scientific research, such as underwater topographic mapping, search and rescue, salvage operations, oil exploration and underwater threat detection, these studies play a key role. At the same time, the forward-looking sonar detector is installed on the remotely operated submersible and autonomous underwater vehicle, which provides strong technical support for the research of underwater target recognition. However, due to the complex and everchanging underwater environment, there may be problems such as signal attenuation distortion, high cost of signal acquisition and transmission, and poor detection accuracy, resulting in low resolution of the obtained sonar images and blurred effective features. In the past decades, the traditional underwater target detection methods are mainly used for sonar image analysis. These methods include detection based on pixel characteristics, gray value or the prior knowledge of the target [1–6]. Due to the complexity of underwater environment, the quality of sonar image is seriously affected by its own noise, reverberation and environmental noise, resulting in low image resolution, unclear edge details, and significant speckle noise. Additionally, considering that the echoes from underwater targets are often weak and their postures are prone to change, the cost of manually obtaining prior information is relatively high. Therefore, traditional sonar image detection methods have many limitations in underwater environments.

To address these issues, researchers have shifted to using deep learning methods to improve underwater target detection. Recently, the depth convolution neural network has shown outstanding achievements in the field of optical image target detection. This development has inspired researchers to apply convolutional neural network technology to sonar image target detection. Fan [7] et al. used the residual network to build a 32 layer backbone network, replacing the Resnet50/101 in Mask R-CNN, and significantly reduced the network’s training parameters without reducing the detection performance, which provides an important reference value for real-time detection and embedded deployment. Similarly, Fan [8] and others optimized the neck module PA-FPN by referring to the idea of adaptive spatial feature fusion module (ASFF) [9], and improved the backbone network in YOLOv4, lightweight the original network, to obtain better feature fusion effect. Zhang [10] and others also optimized YOLOv5 backbone network to improve detection speed. Recently, Zhu [11] and others designed the backbone net-work and detection head in combination with Swin Transformer and DCN, and constructed an anchor free detection model STAFNet, which achieved superior detection performance for three types of targets, namely, victims, ships and aircraft, on the for-ward-looking sonar data set collected by themselves, leading the classic convolutional networks such as Faster R-CNN and FCOS. Of course, in many studies, researchers have basically borrowed ideas from YOLOv3 network [12]. This network is the first time to introduce feature pyramid fusion [13] into the single-stage detection network, realizing multi-scale feature information fusion and expanding its receiving range. However, there is still room for improvement in the network structure. Therefore, YOLOv5 optimized the depth convolution network and feature pyramid, further enhanced the feature pyramid to extract more levels of multi-scale feature information, thus improving the accuracy of detection.

At present, although YOLOv5 network has shown excellent performance in optical image target detection, it is not widely used in acoustic image target detection applications [14]. In addition, the lack of open sonar image data sets in this field also brings difficulties to the application of deep neural networks. Therefore, this paper proposes an improved CCW-YOLOv5 network model based on transfer learning to optimize the loss function of the bounding box. First, in order to deal with the problem of insufficient data sets in the forward looking sonar image target detection task [15], this research adopts the training strategy of transfer learning. Secondly, considering the characteristics of the target in the forward looking sonar image, this study introduces CoordConv [16] to extract features containing coordinate information, which effectively improves the detection accuracy of small targets in the forward looking sonar image. In addition, in view of the difficulty in learning effective features of sonar images, attention mechanism [17] was introduced to increase the Receptive field of learning, obtain more details and inhibit the learning of invalid information. Finally, aiming at the problem that there are many low-quality training samples in the forward-looking sonar image, the original YOLOv5 bounding box loss function is optimized [18]. This article first introduces two popular SOTA models in the field of object detection and the YOLOv5 model with better performance. Then, an improved CCW-YOLOv5 network model was proposed. Through experimental verification and performance comparison, it is found that the improved YOLOv5 model outperforms the original YOLOv5 model in sonar image target detection and other popular target detection models such as YOLOv3 [19], Faster R-CNN [20] showing a higher detection efficiency. Finally, the experimental work of this article is summarized and future work prospects are presented, laying the foundation for future target detection in forward-looking sonar images.

## Related work

We have conducted a detailed study on the benchmark model used in this article, which lays the foundation for improving and optimizing the YOLOv5 algorithm in the next section.

Compared with the most popular SOTA detection models such as Faster R-CNN and YOLOv3, YOLOv5 adopts a lot of innovative designs, including the introduction of new activation function, the use of adaptive convolution, the introduction of packet convolu-tion, etc., which further optimizes the loss function, making YOLOv5 achieve higher ac-curacy than its predecessor YOLOv3 at the same speed. At the same time, YOLOv5 main-tains a relatively high detection accuracy while having smaller model parameters and smaller model file size, making it more suitable for deployment on embedded devices and mobile devices. It also has good applicability for resource limited application scenarios. In addition, the author of YOLOv5 provides a visual training and testing interface, simplifying the experimental and testing process. Meanwhile, YOLOv5 also supports multiple combinations of backbone and head, and can perform fine-tuning on different datasets and tasks, adapting to various application scenarios and requirements. YOLOv5 has a nearly 3-fold increase in detection speed compared to YOLOv3, and also has a significant speed advantage over Faster R-CNN, making it suitable for object detection in real-time scenes with high speed requirements. This improvement is undoubtedly beneficial for target detection in sonar images.

**Fig 1:**
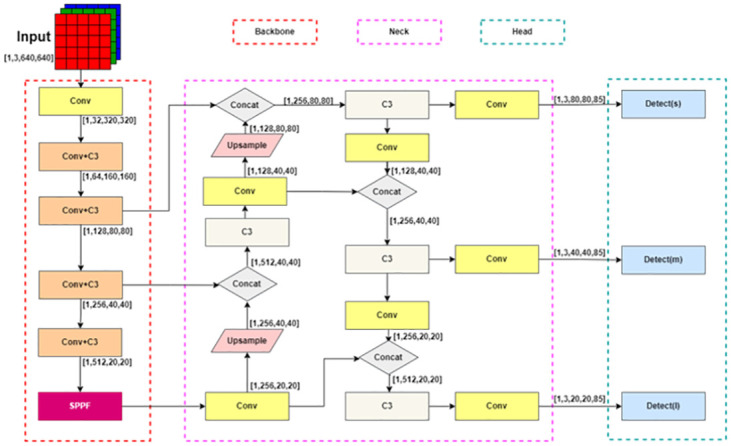
YOLOv5 network model.

Fig 1 depicts the layout of YOLOv5 network model, which consists of three parts: backbone network, Neck and detection head. The input data is initially down sampled five times through the backbone network, and then the last three feature layers are used as the input of the detection head. When data enters the backbone network for the first time, it will first pass through a convolution layer to reduce its width and depth. This step significantly improves the forward propagation speed of the network. After that, the data will pass through four convolution layers and one C3 layer. The C3 layer is an improved version of the CSP layer in YOLOv4 [21], which can extract details more effectively in the backbone network. YOLOv5 model also replaces the activation function of CSP layer according to the improvement of YOLOv4. The 5. x version of YOLOv5 adopts the SPP module proposed in YOLOv3, which can expand the receiving range of the feature layer and effectively fuse features in the Head module. In the 6.0 version of YOLOv5, the SPPF module is added after the last Conv+C3 layer. While maintaining the same calculation results, the calculation speed of this module is twice that of SPP.

In the head section of YOLOv5, the main idea is the same as YOLOv4, and improvements have been made on the basis of PANet. The detection head module of YOLOv5 adopts a multi-level feature fusion method. First, the feature map output from the back-bone network is reduced to the dimension of the feature map by a convolution module, and then the feature maps at different levels are fused to obtain more abundant feature in-formation, so as to improve the detection performance. We focus on the loss function of YOLOv5, whose loss function includes classification loss, confidence loss and regression loss of target prediction. The total loss can be ex-pressed as:

$$\begin{array}{c} Loss_{\text{total}}=\lambda_{1}L_{\text{box}}+\lambda_{2}L_{\text{obj}}+\lambda_{3}L_{\text{cls}} \end{array}$$ (1)

where L_box_, L_obj_ and L_cls_ represents target regression loss, target prediction confidence loss, and category loss, respectively. Under the limitations of the optimizer, it is necessary to increase the corresponding gain for each loss in object detection to scale and balance each loss [22]. In YOLOv5, three gains were determined through multiple experiments λ_1_, λ_2_ and λ_3_ are 0.05,1 and 0.5, respectively. By adding gain and summation to each type of loss, the loss of the image can be obtained from the output of the feature scale.

A large number of experimental results indicate that these networks have good performance in the current field of optical object detection. However, in the application of sonar image object detection, they are often overlooked. Therefore, this article uses these object detection networks to conduct research experiments on object detection in forward looking sonar images, and improves and optimizes the YOLOv5 network to better adapt to forward looking sonar images.

## Materials and methods

This part proposes an improved YOLOv5 target detection algorithm, The model includes transfer learning, improved loss function algorithm of boundary box regression (BBR), CoordCbam-YOLOv5 network and the overall training process of the improved model.

### Transfer learning

**Fig 2:**
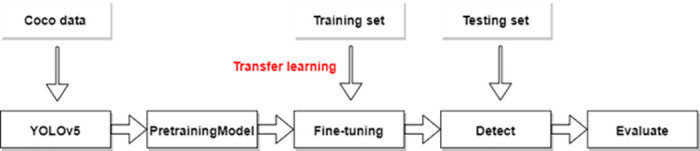
Flow chart of training and testing based on transfer learning.

Due to the difficulty and high cost of underwater experiments, obtaining sufficient and effective sonar image data [23] has become a major challenge. The training and fitting of deep convolution neural networks depend on a large number of training samples. For example, in Pascal VOC [24] and COCO public data sets, YOLOv5 training samples contain 16551 images in 20 categories and 118287 images in 80 categories, respectively. In contrast, the forward-looking sonar image data set available in this study only contains 9200 samples, distributed in 10 categories. In the data set used, the samples of each category are uneven, and the average training samples of the forward-looking sonar image are far less than the other two data sets from the perspective of data volume, which affects the convergence performance of the network. In order to solve the problem of insufficient training samples, this research adopts the transfer learning method, using the YOLOv5 network weight obtained from training on the optical image data set as the pre-training weight, and fine tune it to better adapt to the forward-looking sonar image data set. The research shows that it is effective to use the optical image training model as the acoustic image pre-training model, because the underlying features in the depth network are common in different tasks. We put YOLOv5 on the COCO data set for pre training, and then fine tune it to make it suitable for target detection of forward looking sonar. Then, we test the model with test sets and evaluate the test results. The process is shown in Fig 2.

### Improved loss function algorithm of BBR

Since its inception, the YOLO series of one-stage target detectors have been recognized by most researchers and applied in various scenarios. For example, YOLOv1 constructs a loss function weighted by the regression loss, classification loss and object loss of the bounding box. So far, the construction of the loss function is still the most effective loss function paradigm in target detection tasks [25, 26], in which the boundary box regression loss directly determines the positioning performance of the model. Therefore, in order to further improve the localization performance of the model, it is essential to design a well-designed bounding box regression loss.

For anchor box $\vec{B}=[\begin{array}{llll} x & y & \omega & h \end{array}]$, the values correspond to the center coordinates and size of the bounding box. Similarly, ${\vec{B}}_{\text{gt}}=[x_{\text{gt}}y_{\text{gt}}\omega_{\text{gt}}h_{\text{gt}}]$ describes the attributes of the target box. YOLOv1 and YOLOv2 have similar ideas when defining BBR loss. YOLOv2 defines BBR loss as:

$$\begin{array}{c} L(\vec{B},{\vec{B}}_{\text{gt}})=\|\vec{B}-{\vec{B}}_{\text{gt}}\| \end{array}$$ (2)

Intersection over Union [27] (IoU) is used to measure the degree of overlap between anchor boxes and target boxes in target detection tasks. It effectively shields the interference of boundary box size in a proportional form, allowing the model to use L_IoU_ when IoU (Eq 3) is used as a BBR loss, it can effectively balance the learning of large and small goals.

$$\begin{array}{c} L_{\text{IoU}}=1-IoU=1-\frac{W_{\text{i}}H_{\text{i}}}{S_{\text{u}}} \end{array}$$ (3)

**Fig 3:**
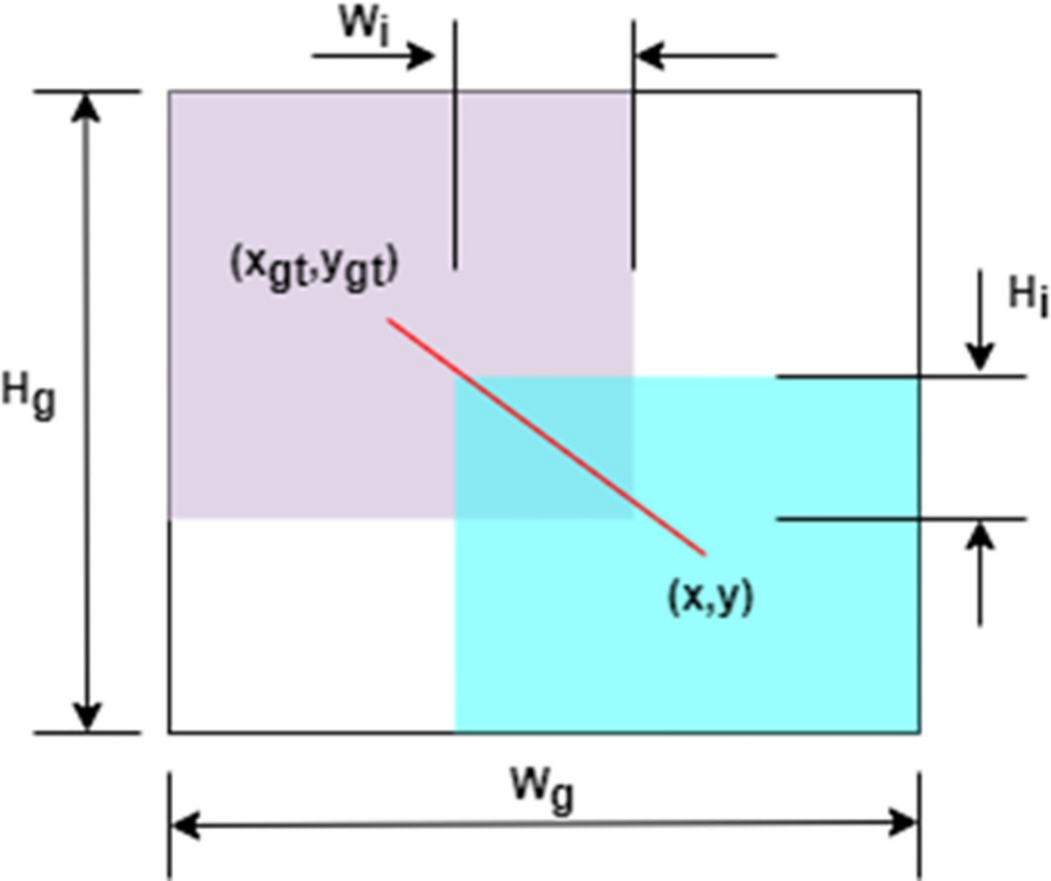
The connection between the smallest bounding box (green) and the center point (red), where the joint area is S_u_ = *ωh* + ω_gt_*h*_gt_ − *W*_i_*H_i_*.

However, L_IoU_ also has a fatal flaw that can be observed from Eq 3. (∂*L*_IoU_)/(∂*W*_i_) = 0 when there is no overlap between the bounding boxes (*W*_i_ = 0*orH*_i_ = 0), *L*_IoU_ Gradient disappearance of IoU backpropagation. Therefore, the width W_i_ of the overlapping area cannot be updated during the training process (as shown in Fig 3).

$$\begin{array}{c} \frac{\partial L_{\text{IoU}}}{\partial Wi}=\{\begin{array}{cc} -H_{\text{i}}\frac{IoU+1}{S_{\text{u}}}, & W_{\text{i}}>0\\ 0, & W_{\text{i}}=0 \end{array} \end{array}$$ (4)

Previous studies [28–31] have considered many geometric factors related to bounding boxes, and the bounding box losses currently used are based on addition losses, and the penalty term R_i_ is constructed to solve this problem. The existing BBR losses follow the following paradigm:

$$\begin{array}{c} L_{\text{i}}=L_{\text{IoU}}+R_{\text{i}} \end{array}$$ (5)

Due to the inevitable inclusion of low-quality examples in the training data of forward-looking sonar images, geometric metrics such as distance and aspect ratio can exacerbate the punishment for low-quality examples, leading to a decrease in the generalization performance of the model. A good loss function should weaken the punishment of geometric metrics when the anchor frame and target frame coincide better, but more intervention training will make the model have better generalization ability. On this basis, distance attention was constructed based on distance measurement, resulting in WIoU_v1_ with a two-layer attention mechanism:

$$L_{{\text{WIoU}}_{\text{v}1}}={\text{R}}_{\text{WIoU}}L_{\text{IoU}}$$ (6)

$$\begin{array}{c} R_{\text{WIoU}}=\left(\frac{{\left(x-x_{\text{gt}}\right)}^{2}+{\left(y-y_{\text{gt}}\right)}^{2}}{{\left(W_{2}^{g}+H_{2}^{g}\right)}^{*}}\right) \end{array}$$ (7)

where *R*(*WIoU*) ∈ [1,e), which will significantly amplify the *L*_IoU_ of the ordinary mass anchor box; L_IoU_ ∈ [0, 1], This will significantly reduce the *R*_WIoU_ of high-quality anchor boxes and significantly reduce their focus on center point distance when the anchor box and target box overlap well. To prevent *R*_WIoU_ generates gradients that hinder convergence, causing *W*_g_, *H*_g_ is separated from the calculation graph (superscript * indicates this operation). Because it effectively eliminates factors that hinder convergence, we did not introduce new metrics such as aspect ratio. Finally, using *WIOU*_v1_ loss function instead of the original loss function and adjusting the penalty term can significantly improve the detection performance of our model.

### CoordCbam-YOLOv5 network

There are some challenges when conducting underwater sonar imaging. Unlike optical devices, the imaging mechanism of sonar leads to attenuation and distortion of acoustic signals in complex underwater environments. In addition, floating objects and particles can increase the multipath effect of sound waves during transmission, resulting in low contrast and blurry edges of targets in sonar images. Another important issue is that due to factors such as long-range detection capability, beam angle resolution, and acoustic scattering, the target echo area in sonar images is relatively small. This further exacerbates the difficulty of locating targets during the detection process. At the same time, the resolution of sonar images is low, and sector images contain less target information and are prone to noise interference. Therefore, how to focus on important features and suppress unnecessary features during feature learning is also one of the problems that we need to solve.

**Fig 4:**
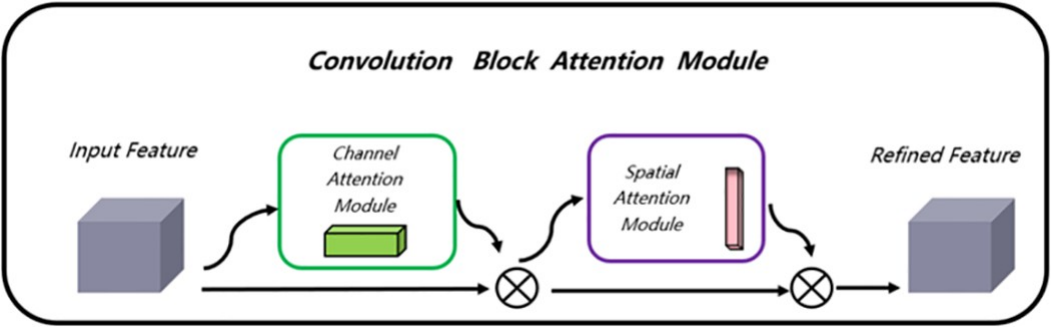
The overview of CBAM.

In response to the above issues, this section proposes to improve the feature extraction module of YOLOv5 network by first entering coordinate convolution and then entering the lightweight attention mechanism module during feature extraction. The addition of coordinate information can not only improve the accuracy of small and medium-sized target detection and positioning regression, but also extract multi-scale high-level features and enrich feature information. The introduction of lightweight attention mechanisms(as shown in Fig 4) helps us suppress unimportant feature information, focus on effective features, and greatly improve model recognition performance.

**Fig 5:**
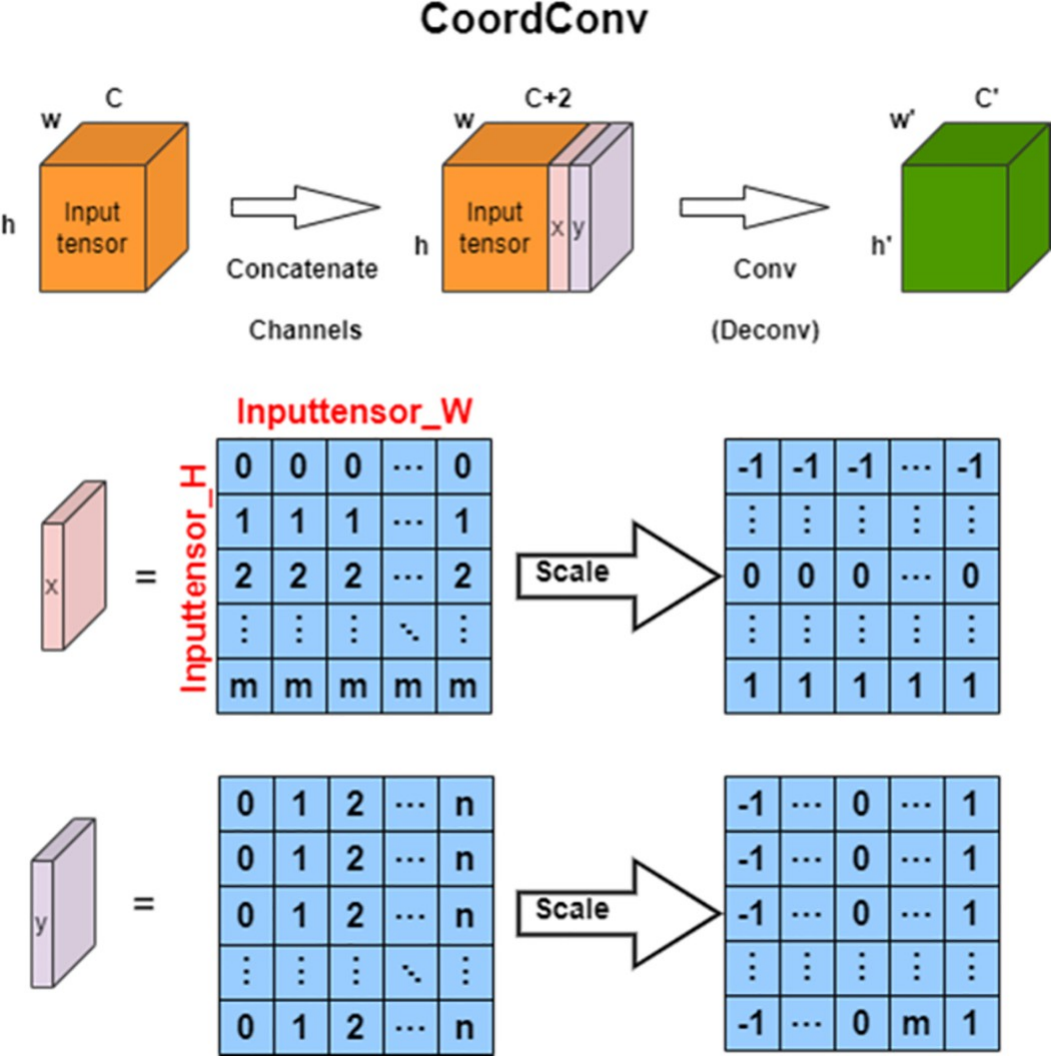
The principle and working mode of CoordConv.

**Fig 6:**
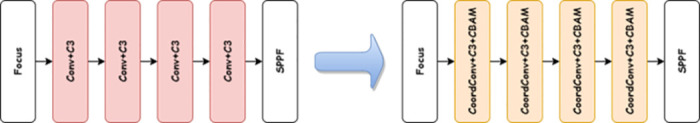
Introduce coordinate information and attention mechanism to YOLOv5 backbone network.

This paper uses the form of coordinate convolution module to expand the convolution layer of the original YOLOv5 backbone network. The detailed description of coordinate convolution is shown in Fig 5. Before extracting parameterized features through convolution each time, we add additional x and y coordinate channel information for each pixel of the input tensor. Due to the different sizes of tensors during each feature ex-traction process, x and y are further scaled to between [-1,1]. The two generated two-dimensional coordinate matrices are processed through convolutional layers, which are transformed into advanced features containing pixel level coordinate information, providing more detailed feature information for the position regression of the detection head. It is worth noting that these extracted advanced features contain coordinate information, which is very beneficial for improving the detection and localization accuracy of small targets in forward-looking sonar images. Considering that the features of forward-looking sonar images are relatively weak, after each regression localization of coordinate information in the backbone network, the system will continue to further enhance feature learning through channel and spatial attention mechanism modules, which significantly enhances the accuracy of target detection in forward-looking sonar images. The specific structure is shown in Fig 6.

### Overall training process of the target mode

**Fig 7:**
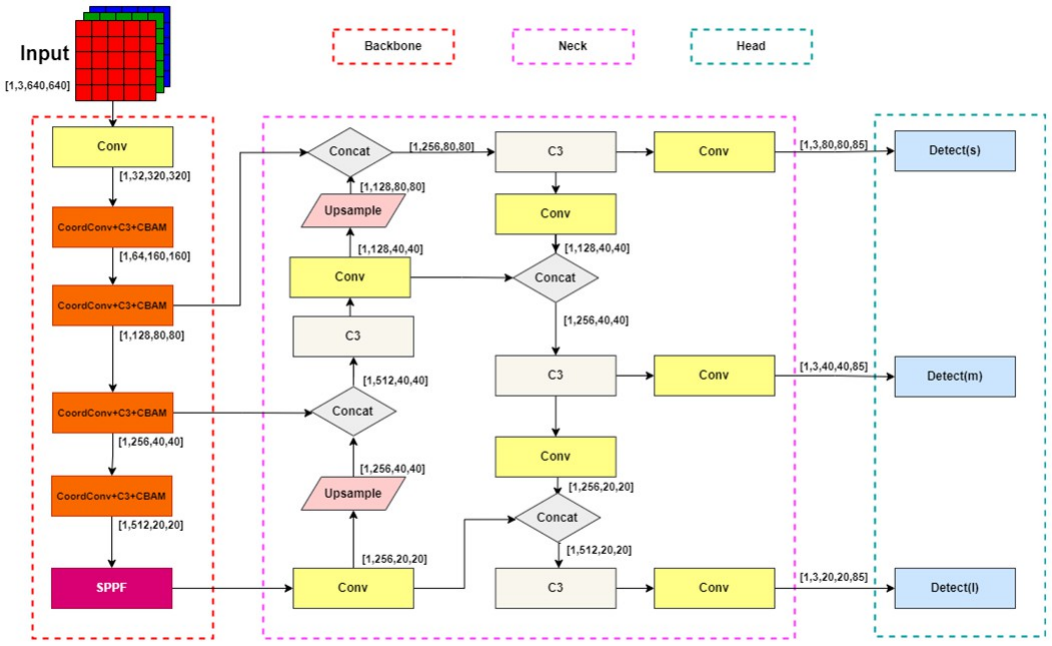
CCW-YOLOv5 network architecture diagram.

In this section, we have provided a detailed explanation of the steps and training process of the proposed target model. The improved CCW-YOLOv5 network structure diagram is shown in Fig 7.

**Fig 8:**
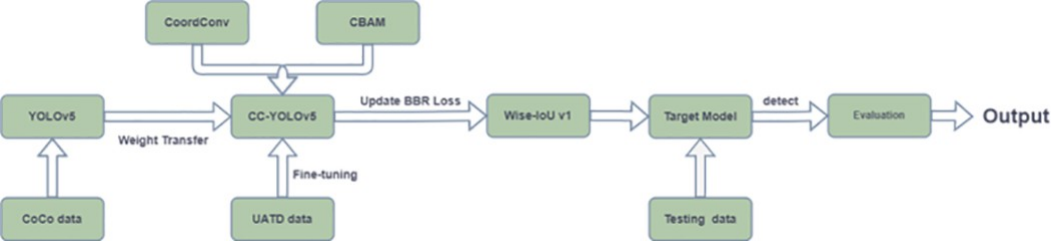
Target model improvement, training, and testing flowchart.

The specific steps and training process are shown in Fig 8, firstly, we added feature coordinate information to the feature extraction module and convolved it with the coordinate information to improve the regression accuracy of localization. Subsequently, an attention mechanism module was introduced to enhance the learning effectiveness of features. Secondly, we optimized the original boundary box loss function. Subsequently, transfer learning methods were used to train the model and fine tuned to achieve optimal training performance. The test set is used to test the target model. Finally, we evaluated the performance of the detection results and identified key performance indicators such as mAP and FPS for the model.

## Results and discussion

### Dataset introduction

The dataset used in this article is the open-source Underwater Acoustic Target Detection (UATD) dataset [32], available at https://figshare.com/articles/dataset/UATD_Dataset/21331143. This dataset consists of over 9000 multi beam forward looking sonar (MFLS) images captured using the Tritech Gemini 1200ik sonar. This dataset provides raw data for sonar images, collected from lakes and shallow water, consisting of ten types of target objects: cubes, spheres, cylinders, human models, tires, circular cages, square cages, metal barrels, aircraft models, and ROVs. Among them, the echo intensity data is stored in the first channel of the BMP image file, and the data in the other two channels of the image is the same as the first channel. The accompanying XML annotation file can be divided into four parts, namely “sound”, “file”, “size”, and “object”. This dataset was launched by the Tiger Whale Open-Source Program, which aims to build an open experimental platform, aiming to improve the cur-rent conditions for underwater research, lower the threshold for underwater research, and promote the development of underwater research.

### Experimental environment and parameter settings

The experiment in this article was conducted in a PyTorch 1.7.0 Python 3.8 (ub-untu18.04) Cuda 11.0 environment with a 12 vCPU Intel (R) Xeon (R) Platinum 8255C CPU @ 2.50GHz and an RTX 3080 (10GB) GPU. The experiment uses YOLOv5 v6.0 version. In the experiment, different models are trained and tested based on the transfer learning method, and the performance of the algorithm is compared and analyzed. This experiment divides the dataset of each algorithm into training, validation, and testing sets according to the proportion provided by the original dataset (7600:800:800) to ensure the authoritative comparison of algorithm results. Several important parameter settings in this chapter’s experiment are shown in Table 1.

**Table 1:** Parameter settings.

Experimental parameters	Set Value
*lr*0	0.005
*lrf*	0.1
*batch*_*s*_ *ize*	8
*linear*_*l*_ *r*	False
*optimizer*	Adam
*imgsz*	640

### Test performance evaluation index

In this study, we use mAP (average of average accuracy) and FPS (frames processed per second) as indicators to evaluate the performance of each network in target detection of forward looking sonar images. MAP first appeared in the PASCAL Visual Objects Classes (VOC) contest, and its definition is as follows:

$$\begin{array}{c} A_{\text{p}}=\frac{1}{11}\sum_{R\in \left\{0,0.1,\ldots ,1\right\}}P_{\text{interp}}\left(R\right) \end{array}$$ (8)

$$\begin{array}{c} mAP=\frac{1}{N}\sum_{i=1}^{N}AP_{\text{i}} \end{array}$$ (9)

where P and R represent the accuracy and recall rates of each object respectively. In order to obtain the precision recall curve, the prediction results of the model must be ranked first (in descending order of confidence of each prediction value). Because they are ranked according to confidence, 11 different confidence thresholds are actually selected. Then, AP is defined as the average value of precision under the 11 recall, which can represent the whole precision recall curve (area under the curve) AP_i_ is the AP value of each category i, and N is the total number of categories.

In the image target detection task, FPS represents the number of images inferred by the model per second. Only fast reasoning speed can realize real-time detection, which is very important in some application scenarios.

$$\begin{array}{c} FPS=\frac{1000(ms)}{pre-process=interferce+NMS} \end{array}$$ (10)

where pre-process is the image pre-processing time, including image keeping aspect ratio scaling and padding, channel transformation and dimension raising processing; Input is the reasoning speed, which refers to the time from the image input model after pre-processing to the model output results; NMS refers to post-processing time, such as line con-version of model output results.

### Result analysis

**Fig 9:**
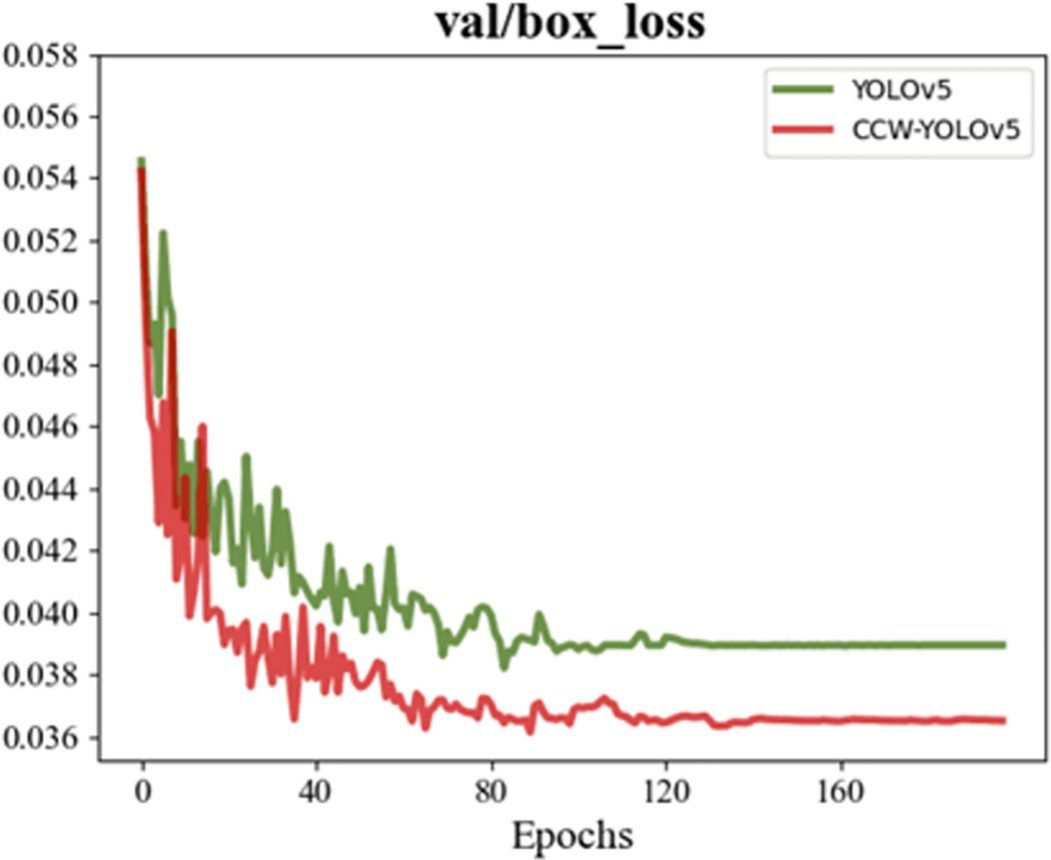
Loss between prediction box and anchor box during training.

**Fig 10:**
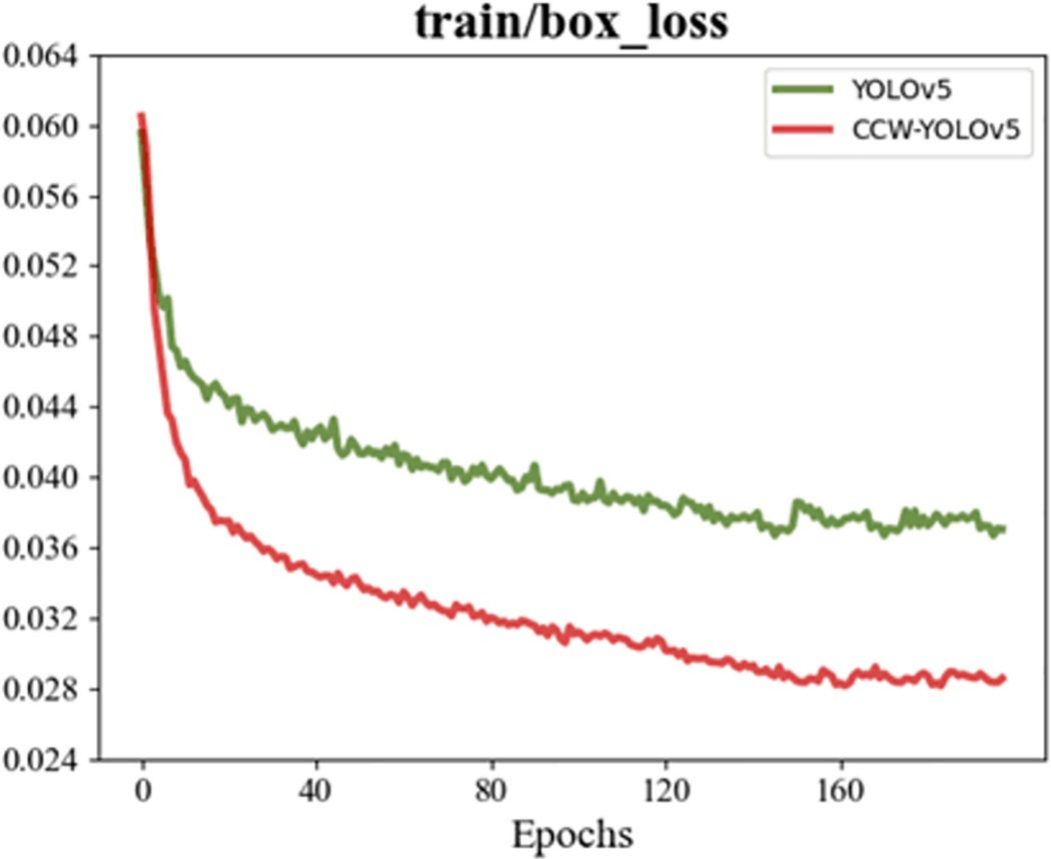
Loss between prediction frame and anchor frame during verification.

Fig 9 is the loss function of the boundary box of the training process and verification process before the improvement, and Fig 10 is the loss function of the boundary box of the training process and verification process after the improvement. The box_loss of the training set and verification set before and after the improvement shows that the loss of the initial training decreases sharply, which indicates that the model learned a lot of information at the initial stage. With the increase of training period, the improved loss oscillation becomes weaker and weaker, and finally stabilizes at about 0.027, lower than the loss before improvement. It can be clearly seen that the volatility of the improved box_loss is smaller when it tends to converge, and the improved training and verification box_loss is significantly smaller than that before improvement.

**Fig 11:**
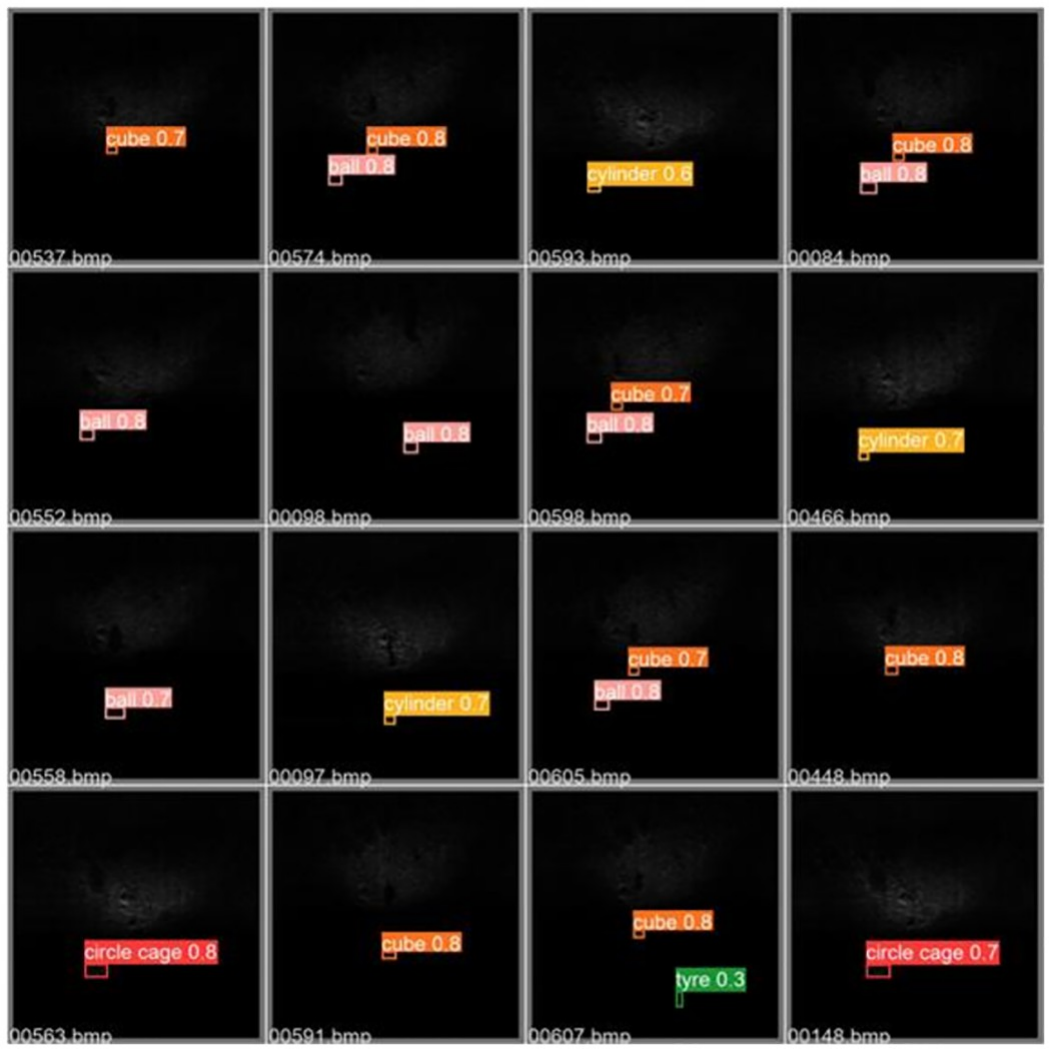
CCW-YOLOv5 model prediction effect diagram.

In addition, in order to visually demonstrate the target detection performance of the CCW-YOLOv5 model proposed in this chapter in practical situations, prediction results of various targets in multi beam forward looking sonar images were selected from the UATD validation set, as shown in Fig 11.

**Fig 12:**
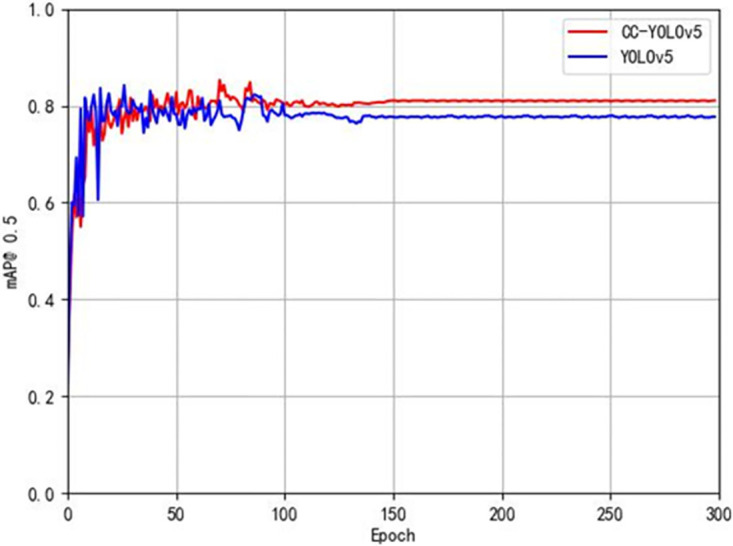
Comparison of validation set mAP test curves.

**Fig 13:**
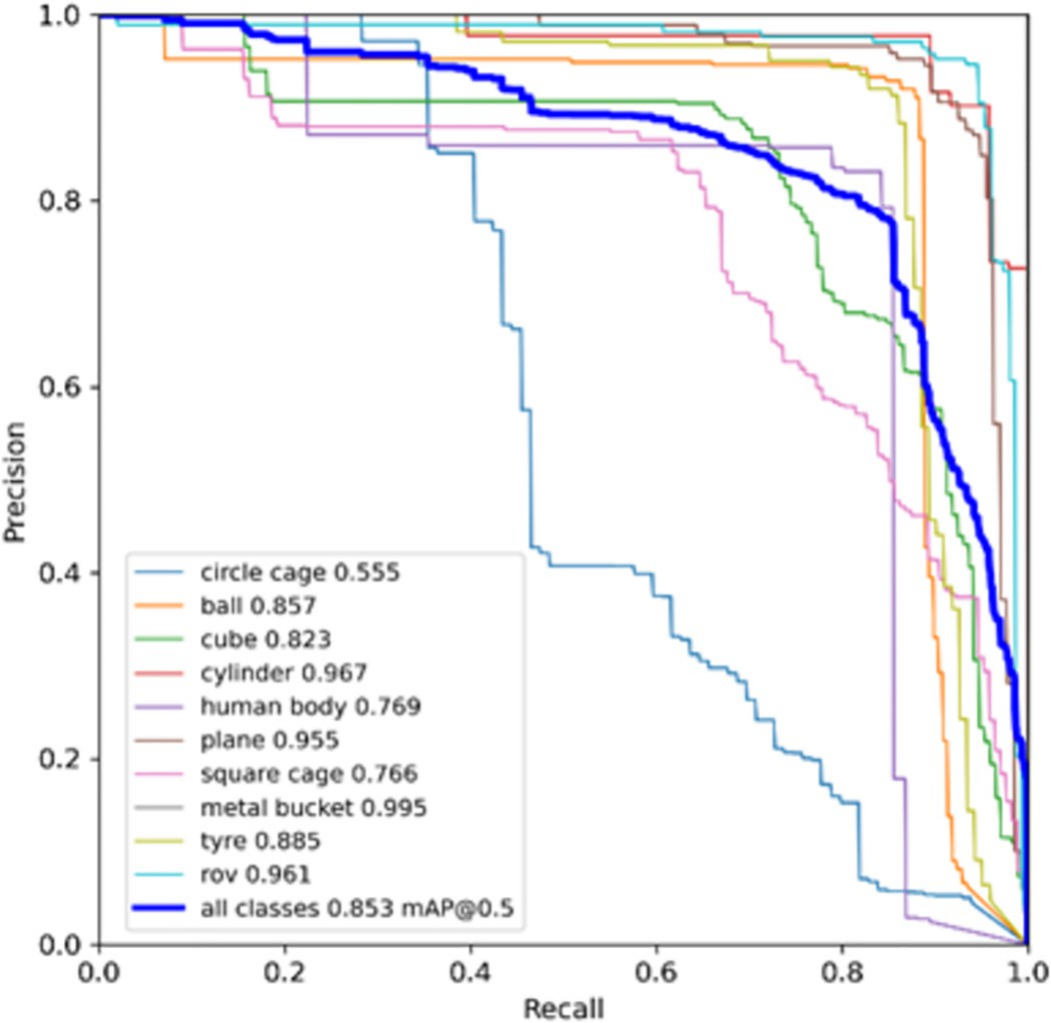
CCW-YOLOv5 P-R curve.

**Fig 14:**
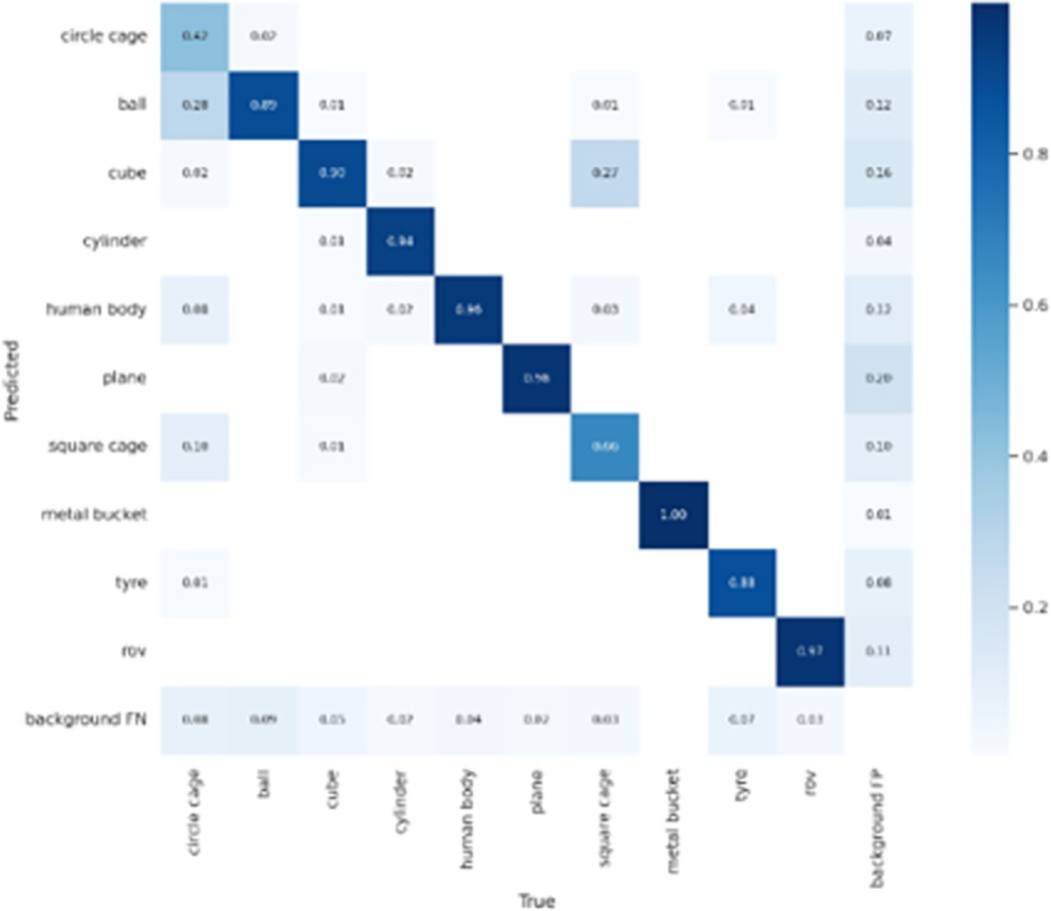
Confusion matrix of test results for CCW-YOLOv5.

Fig 12 shows the improved CCW-YOLOv5 model and the baseline YOLOv5 model map@0.5 After the verification set is stable, the comparison chart is displayed in the map@0.5 In terms of indicators, the improved CCW-YOLOv5 is 3.6% higher than the baseline model. The comparison of the accuracy of the two training results shows that the improved model has certain advantages in improving the detection accuracy of forward-looking sonar images. Fig 13 shows the change trend of accuracy and recall of 10 types of target detection using forward-looking sonar. All categories map@0.5 It reached 85.3%. In addition, as shown in Fig 14, we visualized the results of different categories of targets detected by the CCW-YOLOv5 model in the form of confusion matrix.

The detection results of different types of targets show that CCW-YOLOv5 performs well in the detection of various targets. However, the background noise in the forward looking sonar image sometimes causes the network to incorrectly recognize some background areas as targets. Therefore, the research on sonar image denoising is worthy of our subsequent focus.

Finally, we trained and tested the two most popular target detection models and the original YOLOv5, and comprehensively compared the performance of these models. As shown in Table 2.

**Table 2:** Target detection results of each model in forward looking sonar images.

Model	Backbone	mAP@0.5	Params	FLOPs	FPS
*FasterR* − *CNN*	Resnet-18	0.839	28.17M	49.78G	44.1
*FasterR* − *CNN*	Resnet-50	0.829	41.17M	63.29G	32.9
*FasterR* − *CNN*	Resnet-101	0.818	60.16M	82.77G	26.6
*YOLOv*3	Darknnet-53	0.801	61.57M	49.67G	49.8
*YOLOv*3	MobilenetV2	0.787	3.68M	4.22G	93.4
*YOLOv*5	CSPDarknet-53	0.815	7.03M	15.8G	83
*YOLOv*7	ELAN-Net	0.853	36.9M	104G	16
*YOLOv*8	CSPDarknet-c2f	0.856	11.1M	28.6G	49
**CCW-YOLOv5**	CSPDarknet-53	**0.853**	**7.75M**	**17.1G**	**54**

After comparing the performance of different models in training and testing, our method performs well in forward looking sonar image object detection, achieving 85.3% accuracy map@0.5 Detection accuracy. In addition, the model also performs well in terms of efficiency, with a parameter count of 7.75M Params and 17.1G FLOPs, and a maximum inference speed of 54 FPS on the local machine. Compared with the original YOLOv5 network, our CCW-YOLOv5 network based on transfer learning maintains efficiency and parameter quantity unchanged, map@0.5 Improved by 4.2%.

In addition, by comparing the experimental results of Faster R-CNN and YOLOv3 with different backbone networks, it can be seen that Faster R-CNN with Resnet-18 backbone network is the best mAP@0.5 The value is 83.9%. However, its network structure is too complex, the number of parameters and floating numbers are too large, and its efficiency is far lower than that of the CCW-YOLOv5 model proposed in this paper. On the other hand, YOOv3 with MobilenetV2 backbone performs well in efficiency, only 3.68M Params and 4.22G FLOPs. The fastest reasoning speed tested on the local machine is 93.4 FPS. However, the detection accuracy has not achieved satisfactory results.

Meanwhile, we also compared the performance of Faster R-CNN and YOLOv3 using different backbone networks. Resnet-18, as the backbone network of Faster R-CNN, achieved a peak of 83.9% map@0.5 However, its network structure is complex, with a large number of parameters and floating-point numbers, and its efficiency is far inferior to the CCW-YOLOv5 model. Although the YOLOv3, which is the backbone of MobilenetV2, performs well in efficiency with parameters of 3.68M Params and 4.22G FLOPs, and the highest inference speed on the local machine is 93.4 FPS, its detection accuracy has not reached the ideal level. In the target detection task of forward-looking sonar images, the single-stage detection network model like YOLOv5 performs much better than the two-level detection model. This is because YOLOv5 can directly perform regression calculation on regions without first identifying candidate regions. On the contrary, Faster R-CNN and other two-level detection models need to identify candidate regions first, and then analyze these regions, resulting in their lower efficiency than single-stage network models.

Finally, this article will also test the more popular YOLOv7 and YOLOv8 locally, although their detection accuracy is excellent and the best mAP@0.5 They reached 85.3% and 85.6% respectively, but due to their complex network structure, their Params and FLOPs are much larger than CCW-YOLOv5 by more than 1.5 times, which has a fatal impact on the real-time requirement of forward-looking sonar image object detection tasks.

These experimental results indicate that our proposed CCW-YOLOv5 network has good performance in forward looking sonar image object detection tasks, providing valuable reference for deep learning based research on forward looking sonar image object detection.

### Ablation experiment

To verify the effectiveness of our proposed structure and techniques, we conducted ablation studies on the UATD dataset. As shown in Table 3, first of all, we have improved the coordinate convolution, CBAM, and *WIOU*_v1_ loss function respectively on the basis of the original YOLOv5 6.0 network structure. Under the influence of the attention mechanism, the recognition accuracy of the model has the highest increase among the three, mAP@0.5 Improved by 2.6% compared to the original YOLOv5 network. This indicates that adding appropriate attention mechanisms can effectively help the model focus on the target and achieve more accurate classification recognition. However, a single structural split cannot prove the optimality of our model, so we combined the proposed optimization improvement in pairs. The results indicate that despite the combined effects of coordinate convolution and attention mechanism, mAP@0.5 We achieved 84.7 and achieved a good 82% F1 score, but in comparison, our proposed CCW-YOLOv5 achieved better detection performance.

**Table 3:** Results of ablation research on various tricks.

Model	mAP@0.5	mAP@0.5:0.95	F1
*YOLOv*5 + *CoordConv*	0.81	0.357	0.85
*YOLOv*5 + *CBAM*	0.841	0.36	0.82
*YOLOv*_5_ + *WIoU*_v_1	0.82	0.361	0.81
*YOLOv*5 + *CoordConv* + *CBAM*	0.847	0.346	0.82
*YOLOv*5 + *CoordConv* + *WIoU*_v_1	0.836	0.342	0.83
*YOLOv*5 + *CBAM* + *WIoU*_v_1	0.841	0.34	0.82
**CCW-YOLOv5**	0.853	0.339	0.83

## Conclusion

In this study, we explore an improved CCW-YOLOv5 algorithm specifically for target detection in multi-beam forward looking sonar images. Considering the difficulty of target identification in forward-looking sonar images, especially for small or partially obscured objects, we developed a CoordCbam-YOLOv5 detection model. In addition, an improved WIOUv1 loss function is introduced to deal with the problem of low quality training samples commonly found in forward-looking sonar images. First, we adjust the original YOLOv5 model by transfer learning to make it more suitable for target detection in forward-looking sonar images.

In the experimental stage, we trained and tested multiple convolutional network models such as Faster R-CNN, YOLOv3-Darknet-53, YOLOv3-MobilenetV2, YOLOv5, YOLOv7 and YOLOv8. After comparative analysis, our CCW-YOLOv5 network based on transfer learning has the best performance in detection accuracy and speed. Specifically, its mAP@0.5 detection accuracy reached 85.3%, and it also outperformed other models in terms of reasoning speed, reaching 54 FPS.

However, due to the complexity of underwater environments and the susceptibility of forward looking sonar images to noise, coupled with the sparse distribution and lack of features of targets in sonar images, as well as the small size of datasets used in existing work, the generalization ability of the model has not been fully verified. To address these challenges, future research will focus on obtaining and annotating forward-looking sonar image data, as well as removing background noise.
